# Complete genome and plasmid sequence, and chemotaxonomic analysis of *Francisella* sp. strain W12-1067, now designated as *Allofrancisella heilbronnii*

**DOI:** 10.1186/s12866-025-04600-5

**Published:** 2025-12-09

**Authors:** Kristin Köppen, Kerstin Rydzewski, Klaus Heuner

**Affiliations:** Working group: Cellular Interactions of Bacterial Pathogens, Centre for Biological Threats and Special Pathogens, Highly Pathogenic Microorganisms (ZBS 2), Robert Koch Institute, Seestraße 10, Berlin, 13353 Germany

**Keywords:** Tularemia, Francisellaceae, Allofrancisella heilbronnii, W12-1067, Plasmid, Genome sequence

## Abstract

**Background:**

The zoonotic pathogen *Francisella tularensis* is the etiological agent of tularemia. Tularemia in humans is mainly caused by *F. tularensis* subspecies *tularensis* and *holarctica*, but beneath some opportunistic *Francisella *species, like *F. novicida* and *F. philomiragia,* further genera of the family *Francisellaceae* have been described, such as *Allofrancisella, Parafrancisella* and *Pseudofrancisella*. Less is known about these genera. In the presented study we describe a German *Allofrancisella* isolate which was originally described as *Francisella* sp. strain W12-1067.

**Results:**

Phylogenetic analysis and chemotaxonomy revealed that *Francisella* sp. strain W12-1067 is a separate species of the genus *Allofrancisella* and therefore was named *A. heilbronnii*. *A. heilbronnii* shows several genotypic and phenotypic differences to other *Allofrancisella* species, in growth, LPS and fatty acid composition. The genome size is 1,706,635 bp. A plasmid (6,872 bp) was found within the genome of *A. heilbronnii*, which is designated as pFW12. It encodes six open reading frames including a replication initiation protein and an *iglD* (*tssk*). We showed that pFW12 is transferable and stable in other *Francisallaceae* species, such as *F. tularensis* ssp. *holarctica*, *F. novicida* and *A. inopinata*.

**Conclusion:**

*Francisella* sp. strain W12-1067 belongs to the genus *Allofrancisella *and now is designated as *A. heilbronnii*. In this work we present the annotated, complete genome and plasmid sequence. The genus *Allofrancisella* seems to be the only member of *Francisellaceae* which is negative for the genes of the major virulence island named *Francisella* pathogenicity island (FPI). A putative cryptic plasmid, pFW12, was found in *A. heilbronnii*, which is transferable and stable into other *Francisallaceae* species. Therefore, pFW12 could be used as a *Francisellaceae*-specific vector.

**Supplementary Information:**

The online version contains supplementary material available at 10.1186/s12866-025-04600-5.

## Background

**Fig. 1:**
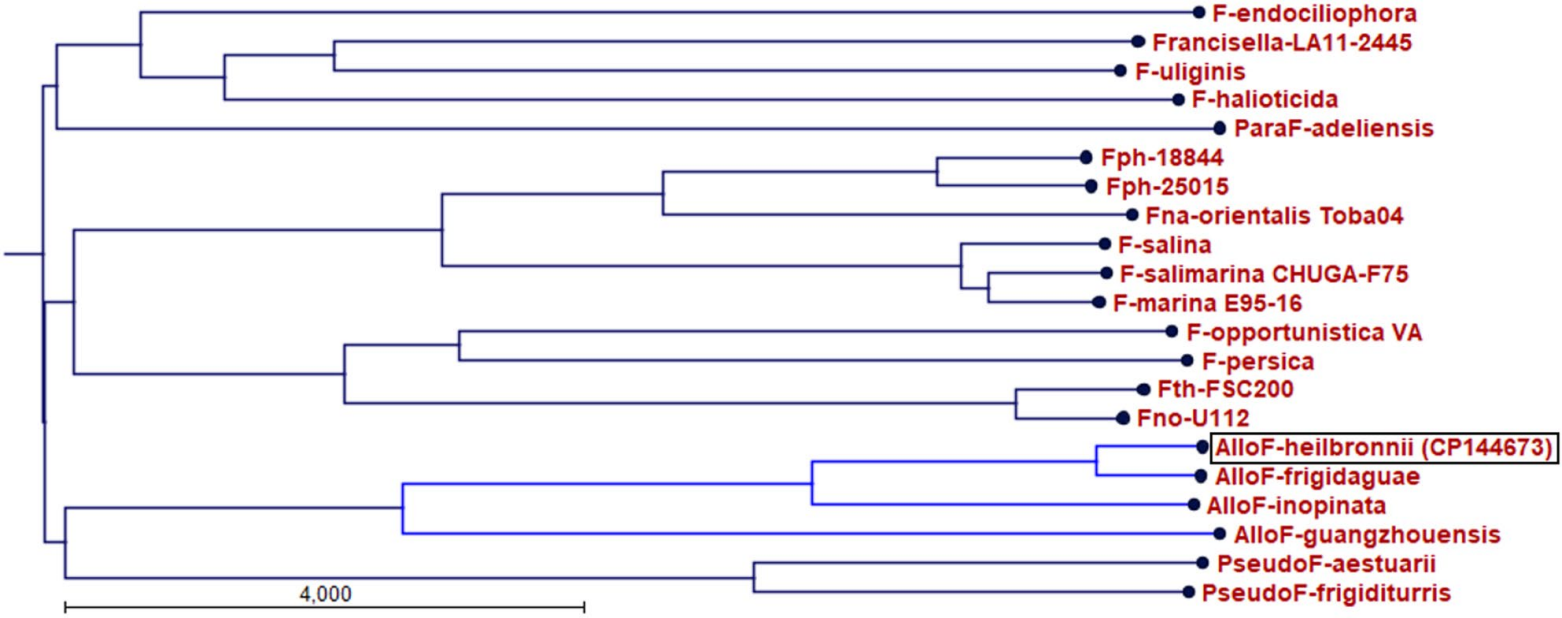
Whole genome alignment tree of *Francisellaceae*. Whole genome sequences were aligned (ANI alignment) and a Neighbor-Joining tree was generated using CLC Genomics Workbench. The tree shows that strain W12-1067 belongs to the genus *Allofrancisella* and is closely related to *A*. *frigidaquae.* Representative genomes of *Francisellaceae* strains: *F. tularensis* ssp. *holarctica* (FSC200, CP003862), *F. novicida* (Fno-U112, NC_008601), *F. opportunistica* (CP022377), *F. persica* (CP013022), *F. philomiragia* (Fph 18844, CP010019), *F. noatunensis* ssp. *orientalis* (CP003402), *F. salimarina* (CP076680), *F. endociliophora* (CP009574), *F.* sp. LA11-2445 (CP041030); *F. halioticida* (CP022132), *F. uliginis* (CP016796), *Allofrancisella* (AlloF) *frigidaquae* (CP038017), *A. guangzhouensis* (CP010427), *A. inopinata* (CP038241), *A. heilbronnii* (CP144673), *Pseudofrancisella* (PseudoF) *frigiditurris* (CP009654), *P. aestuarii* (GCA_003574475.2) and *Parafrancisella* (ParaF) *adeliensis* (CP043424) were used for the alignment

The family *Francisellaceae* is composed not only by the genus *Francisella*, recently also species as *Allofrancisella* spp., *Parafrancisella* spp. and *Pseudofrancisella* spp. were described to be members of this family [1–3]. *F. tularensis* is the causative agent of tularemia, can infect humans and animals and is found in a wide range of wild animals. The most pathogenic species is *F. tularensis* ssp. *tularensis* and is more frequently associated with tularemia cases in the U.S.A [4]. *F. tularensis* ssp. *holarctica* is found in the U.S.A and in the whole northern hemisphere and is the causative agent of tularemia cases in Germany [4–7]. *F. tularensis* ssp. *mediasiatica* is found in Central Asian region (Kazakhstan, Uzbekistan) and Russia (Siberia) [8–10]). There are also many additional species of the genus *Francisella*, such as *F. novicida*, *F. hispaniensis*, *F. philomiragia*, *F. salimarina* and others (see Fig. 1). Some of these species are opportunistic bacteria which are able to cause infections in immunocompromised humans and/or are known to be pathogenic for animals [11–14].

Strains of the genus *Allofrancisella* were described in China and Germany in 2013 and 2014 [15, 16], including different species (*A. guangzhouensis* strain 08HL01032 [CP01427], *A. frigidaquae* strain SYSU 10HL1970 [CP38017] and *A. inopinata* strain SYSU YG23 [CP038241], all three were obtained in China) (see Fig. 1). In 2012 the environmental *Francisella* sp. isolate W12-1067 was found in a cooling tower in South Germany. So far, only a draft genome has been published, but it reveals great similarities to Chinese *Allofrancisella* strains also found in water systems. Even though not all *Francisella*-specific virulence genes are present within the draft genome of this strain, like the *Francisella* pathogenicity island, we experimentally identified virulence-associated genes like the GTP-phosphokinase RelA and the metalloprotease FtsH [16, 17]. Also, we have shown, that the strain is able to persist in human lung explant [18]. The most important virulence factor of *Francisella* is a type six secretion system (T6SS) encoded by the *Francisella* pathogenicity island (FPI) [19]. The originally FPI is not found in strain W12-1067, but two FPI-like islands encoding putative alternative T6SSs are present [16]. Both islands exhibit a significant similarity to the FPI II found in *F. novicida*. However, little is known about their role.

For the genus *Parafrancisella* only one species (*P*. *adeliensis*) has been described in 2019 [2] and yet two species are known for the genus *Pseudofrancisella*,* P. frigiditurris* (published as *F. frigiditurris* [1, 20]), and *P. aestuarii* [2]. To our knowledge it is yet not known if these species may cause infections in animals or humans.

Plasmids are important vehicles of genetical material. In *F. tularensis* ssp. *holarctica* a plasmid is commonly absent, but in some other species of *Francisellaceae* (cryptic) plasmids are present, such as *F. philomiragia*, *F. novicida*, *F. frigiditurris*, *F. opportunistica*, *F. hispaniensis* and *A. guangzhouensis* [21]. Less is known about the function or role of plasmids in *Francisellaceae*. Their sizes range between a few thousand base pairs (bp) with only one ORF to more than 40,000 bp containing more than 50 ORFs [21]. Some of the plasmid-encoded genes are putatively involved in replication and conjugative transfer. Still, there is no experimental evidence for a conjugative transfer of a *Francisella*-derived plasmid.

In this work, we generated a complete, annotated version of the genome sequence of *Francisella* sp. isolate W12-1067 using our previously published draft version [16] and by the re-sequencing its whole DNA using Illumina and Nanopore sequencing methods. Thereby, we identified a plasmid, which we also describe in this study. Further phylogenetic analysis and chemotaxonomy revealed that this strain belongs to the genus *Allofrancisella* and we classified strain W12-1067 as a new species, named *A. heilbronnii*.

## Methods

### Bacterial strains and growth conditions

*Francisella tularensis* ssp. *holarctica* Live Vaccine Strain (LVS), *F. novicida* U112, *A*. *guangzhouensis* (DSM 102975), *A. inopinata* (DSM 101834), *A. frigidaquae* (DSM 101835), *A. heilbronnii* (DSM 118443) [16] and *A. heilbronnii* Scatter Clone #64 and the derivative strains were grown in medium T [22, 23] or on agar plates consisting of medium T, hemoglobin and charcoal (MTKH plates [24]), at 37 °C. *A. heilbronnii* Scatter Clone #64 (Sc#64) was obtained using Tn5 mutagenesis and a virulence screening test with *Acanthamoeba lenticulate* (scatter screen, for further information see [17]). Sc#64 possesses a plasmid with the Tn5 sequence integrated within the plasmid-encoded FRA_07945 (resolvase). *Escherichia coli* DH5α and derivative strains were grown in Luria broth (LB) or on LB agar plates at 37 °C. Kanamycin was used for *Francisellaceae* at the concentration of 10 µg/ml (in liquid medium) and 20 µg/ml (MTKH plates) and for *E. coli* at a concentration of 40 µg/ml.

Bacterial growth (biomass, culture density) was measured by Cell Growth Quantifier (CGQ, Aquila BioLabs, Beasweiler, Germany). Volumes of 20 ml of medium T were inoculated with an over-night culture of the *Allo*f*rancisella* strains leading to a final OD_600_ of 0.3. Culture flasks were incubated at 37 °C and 250 rpm to determine backscatter values (as “scattered light intensity”) automatically every hour of incubation by CGQ [25].

### Phenotypic and biochemical characterization

Antibiotic susceptibility testing (inhibition zone in mm) was performed on MTKH agar plates at 37 °C after 3 d of incubation (no AST standard). For biomass production the strain was cultivated in liquid medium T at 28 °C for 3 days. Analysis of respiratory quinones, cellular fatty acids, polar lipids, as well as antibiotic susceptibility testing and API Zym assay were carried out by DSMZ Services, Leibnitz-Institut DSMZ - “Deutsche Sammlung von Mikroorganismen and Zellkulturen GmbH, Braunschweig, Germany”.

### Western blot analysis

LPS detection was carried out by sodium dodecyl sulphate-polyacrylamide gel electrophoresis (SDS-PAGE) and Western blotting. The SDS-PAGE assay was performed as described previously [26]. Equal amounts of aliquots of *Francisella* strains were boiled for 10 min in Roti Load 1 buffer. A total of 25 µl of the solution was loaded onto a 12% SDS polyacrylamide gel. Western blotting was carried out using a polyclonal anti-LPS protein A purified antibody generated against isolated LPS from *F.* sp. strain W12-1067 in rabbits (AB016013-1, LifeTein, LLC, Hillsborough New Jersey, USA) diluted in 1% milk–Tris-buffered saline (TBS) (1:1000). A horseradish peroxidase-conjugated goat anti-rabbit antibody was used as secondary antibody (1:1000) (Invitrogen, # 32460, Fisher Scientific, Germany). Visualization was done by incubation of the blot with 50 ml of color reaction solution (47 ml of 1x TBS, 3 ml of 4-chloro-1-naphthol, and 80 µl of H_2_O_2_), and the reaction was stopped with distilled water.

### DNA preparation, PCR analysis and Sanger sequencing

For genomic DNA isolation the DNeasy Blood & Tissue Kit (Qiagen, Hilden, Germany) was used and for plasmid DNA isolation the GeneJEt Plasmid Midiprep-Kit (Thermo Fisher Scientific, Germany) was used, both according to manufacturer’s instruction. DNA concentration was measured with Qubit (Thermo FisherScientific, Gemany) and used for subsequent analysis (PCR, sequencing). All plasmids and primers used in this study are listed in Tables 1 and 2. Conventional polymerase chain reaction (PCR) was carried out using a Thermocycler TRIO-Thermoblock (Biometra, Göttingen, Germany). The Q5 DNA polymerase (New England Biolabs, Massachusetts, US) was used to generate the mutated DNA constructs and the Taq DNA polymerase to screen the obtained mutant clones. For Q5 the initial denaturation was performed at 98 °C for 30 s and final extension was performed at 72 °C for up to 5 min. The cycling conditions (35 cycles) were 98 °C for 10 s, 59 °C for 30 s and 72 °C for 30 s per 1 kb. For the Taq DNA polymerase following cycler conditions were used: initial denaturation 94 °C for 2 min, 94 °C for 1 min, 58 °C for 1 min, 72 °C for 1 min per 1 kb and the final extension 72 °C for 5 min. For all PCR reactions ∼ 100 ng of template DNA was used. Oligonucleotides were obtained from Eurofins MWG Operon (Ebersberg, Germany). Obtained PCR product were used for Sanger sequencing (LI-COR-DNA4000; MWG-Biotech, Ebersberg, Germany).

**Table 1:** Plasmids used in this study

Plasmid name	size (bp)	relevant feature
pFW12	6,872	native plasmid
pFW12-Sc64	8,093	Tn5 insertion into resolvase, obtained from Sc#64
pFW12-Sc64 ∆ISFw3	5,585	deletion of both ISFw3 elements
pFW12-Sc64 ∆ori	7,988	deletion of the putative origin of replication
pFW12-Sc64 ∆ISFw3 ∆ori	5,480	deletion of both ISFw3 elements & the putative Ori

**Table 2:** Primer used in this study

Primer name	Primer sequence (5’ ->3’)
delta-ori-F	CACTGTGGAAATAAATGTGTTGAGG
delta-ori-R	TCCCAGCAAACACTTTAATAGCAATG
Contig_71 F	GCTAAACCATCAAATTGCGTTCC
Contig_71 R	CTAATTGCCTTTCATGATTTTGAGCA
P3 R	TCCCAGCAAACACTTTAATAGCA
Contig 71 R F	TGCTCAAAATCATGAAAGGCAAT
Contig 71 F2	TTATAGGGCCTGCTGAGAATAGC
Contig 71 R2	TGCTATTGAGTAGGTCAGTTAAGG
peg 426 F	CAAGAGGTGTCCTTATGCAAGAT
peg 429 R	AACCCCACTAGGACCATCGATAG
Contig 71 F3	AGCTTCATCTACTCTATAAACTCTTGA
Contig 71 R3	TCCCAGAATATAATTTCTCCGTATTCT
IglD F	TGGGAGAACGGTCTGAAATTAGA
iglD R	TCTGAGCCACCTAGCGAAATATG
iglD F R	TCTAATTTCAGACCGTTCTCCCA
iglD R F	CATATTTCGCTAGGTGGCTCAGA
repA F	TGAGATGCCTCAATGCTATTTAGA
repA R	TCTAAATAGCATTGAGGCATCTCA
P1 F	GACCAAAAGTAGCTCAAGATGGC
P2 F	CGGCGTATGGACAACATAGAGTA
P2 R	TACTCTATGTTGTCCATACGCCG
P3 F	TGCTATTAAAGTGTTTGCTGGGA
KAN-2 RP-1	GCAATGTAACATCAGAGATTTTGAG
KAN-2 FP-1	ACCTACAACAAAGCTCTCATCAACC

### Genome sequencing and assembly of a complete genome sequence

DNA was extracted by MagAttract (Qiagen GmbH, Germany) and amplified by REPLI-g MIDI kit (Qiagen GmbH, Germany). The sequencing library was prepared with Nextera XT DNA sample prep kit v3 (Illumina Inc., USA) with 600 cycles. For the MinION sequencing platform, the amplified DNA was endonuclease treated with T7 endonuclease I (New England Biolabs, Ipswich, MA, USA). The whole genome sequencing library was prepared using Oxford Nanopore SQK-LSK108 kit protocol with the native barcoding expansion kit EXP-NBD103 and sequenced on a flow cell with R9.4 chemistry MIN106. No DNA size selection or fragmentation was performed prior to sequencing. Whole genome sequencing was performed on both, MiSeq instrument (Illumina Inc., USA) and MinION instrument (Oxford Nanopore Technologies Ltd, UK), generating 2,951,826 trimmed pair-end read pairs (median read length 225 bp; 35 to 301 bp) and 22,852 long reads (median read length 5398 bp, read length N37 34,290 bp). Illumina reads were trimmed using Trimmomatic (v.0.36) removing low quality bases at the end of reads [27]. Nanopore reads were base-called using Albacore (v2.1.3) (https://community.nanoporetech.com), and adapters were trimmed with Porechop (v.0.2.3_seqan2.1.1). A polished linear sequence was generated with the hybrid assembler Unicycler (v.0.4.7), using both, short Illumina reads (mean coverage 440, plasmid 766) and long Nanopore reads, as input [28]. To generate the final version, the DNA sequences of contigs from accession version AWHF01000000 (BioProject PRJNA214898, BioSample SAMN02315612) as well as Nanopore reads longer than 10 kbp were mapped back to the obtained draft genome sequences using Geneious mapper and a manual inspection was performed. A few ambiguous genome regions were amplified by PCR and sequenced by LI-COR sequencer to confirm identity and correct assembly. Finally, the genome was rotated to start with *dnaA*. Annotation was performed by GenBank using NCBI Prokaryotic Genome Annotation Pipeline (PGAP). The GenBank accession no. of the genome is CP144673 and CP144674 for the plasmid pFW12.

The average nucleotide identity (ANI), calculated by pairwise genome comparison, and phylogenetic analysis (Tree building, Neighpor Joining method) was done by using the CLC Genomics Workbench 24.0.1 software.

### Construction of different forms of pFW12

To generate different forms of the plasmid, the plasmid originated from Sc#64 was used, which was named pFW12-Sc64. For the generation of a plasmid lacking both ISFw3 elements, a PCR was performed with the primer Contig 71 F3 + Contig 71 R3 (PCR product size: ∼ 5.6 kb) targeting the whole pFW12-Sc64 without both ISFw3 elements. The PCR product size was verified by gel electrophoresis and extracted using the Wizard SV Gel and PCR Clean-up System (Promega, Mannheim, Germany). The Q5 generated blunt ends were religated using the T4-DNA-Ligase at 4 °C for over-night and then used for transformation into other *Francisella* strains (see below).

A similar procedure was used to confirm the putative plasmid’s origin of replication. Here the primer pair delta-ori-F and delta-ori-R was used which resulted into a product of ∼ 8 kb when the “native” pFW12-Sc64 was used and to a product size of ∼ 5.5 kb when pFW12-Sc64∆ISFw3 was used as template. Both were proceeded in parallel with the control construct (PCR-product for ∆ISFw3). The same DNA amount of all products (140 ng) was transformed into other *Francisellaceae* strains and resulting clones were counted and the transformation was confirmed by PCR (used primers listed in Table 2). The percentage of positive clones was calculated.

### Bacterial transformation

Plasmid DNA (pFW12 and derivates (see Table 1) was introduced into *Francisellaceae* strains (*F. tularensis ssp. holarctica* LVS, *F. novicida* U112, *A. heilbronnii*, *F. philomiragia* 25017, *A. inopinata*) and into *E. coli* DH5α by electroporation using the Gene Pulser system (Bio-Rad, Munich, Germany). Electroporation was done at 2.5 kV, 600 Ω and 25 µF for *Francisella* and at 1.7 kV, 100 Ω and 25 µF for *E. coli*. After transformation, *Francisella* were incubated in medium T for up to 4 h at 37 °C and then plated onto MTKH agar plates supplemented with kanamycin.

### Plasmid stability test

To test the stability of pFW12-Sc64 and derivates in *F. tularensis* ssp. *holarctica* LVS, the transformants were initially grown over-night in 3 ml medium T supplemented with kanamycin. On the next day, 200 µl of the over-night culture was used to inoculate 3 ml of fresh medium T without kanamycin. Every 12 h, the bacteria were passaged in the same manner. After 10 passages, the bacterial culture was used to measure the OD_600_ and to adjusted to OD_600_ = 1. Bacterial suspension was further diluted and plated onto agar plates with and without kanamycin to determine the number of bacteria (CFU/ml) which still possess pFW12-Sc64 and derivates. Also, plasmid and genomic DNA was extracted and used in PCR analysis to confirmed the presence of the plasmid. Different primer combinations were applied: Contig 71 F/71R, iglD RF/P3R and iglD R/Contig 71RF (see Table 2). Plasmid stability testing was performed in three independent experiments.

## Results and discussion

Strain *A. heilbronnii* W12-1067 (*Ahe*) was isolated from a water reservoir of a cooling tower in Germany [16]. It is a Gram-negative, aerobic, non-motile and non-spore forming pleomorphic bacterium. It was initially named *Francisella* sp. strain W12-1067. It has no homolog of the FPI island, the major virulence factor of *Francisella*, but it exhibits a putative alternative T6SS (aT6SS). The phylogenetic analysis and chemotaxonomy (see below) revealed that *Ahe* belongs to the genus *Allofrancisella*. It shows a relative high ANI value of the genome sequence in comparison to the genome of *A. frigidaquae* (*Afr*). However, based on different genotypic and phenotypic features (see below), we classified strain W12-1067 as a new species (*A. heilbronnii*) within the genus *Allofrancisella*.

### Complete genome sequence, phylogenetic analysis, ANI and DDH values of *A. heilbronnii*

A draft version of the genome sequence of *Ahe* (*F*. sp. isolate W12-1067; contigs: AWHF00000000) was published earlier [16]. In this work, we present the annotated, complete genome sequence (BioProject accession number PRJNA214898, BioSample: SAMN02315612), which has been deposited to GenBank under the accession number CP144673 (chromosome) and CP144674 (plasmid). General genome features, the Average Nucleotide Identity (ANI) and the Digital DNA Hybridization (DDH) values of *Ahe* and further *Allofrancisella* strains are given in Table 3. The 1,706,635 bp circular chromosome of *Ahe* has a GC content of 32.1% with 1,536 protein-coding sequences. The 6,872 bp plasmid has a GC content of 28.9% encoding six genes. This plasmid (pFW-12, for details see below) has been identified by the re-analysis of the genome sequence and was not included in the published draft genome [16]. In addition, we identified two different versions of the major IS-element (ISFw3) to be present at the genome sequence of *Ahe*. ISFw3 (19 copies) and IsFw3* (pseudo-ISFw3, 7 copies) with an insertion of three nucleotides (position 514–516; 5’-TAT-3’) and a frame-shift mutation near the 3’-site of the IS481-like element (see Figure S1). Our analysis revealed that a homolog of ISFw3 seems to be present in *F. opportunistica* (data not shown). The direct comparison of the *Ahe* with the *Afr* chromosome sequence (data not shown) indicated nearly co-linear chromosomes, but *Afr* has a 32,455 bp smaller chromosome and it exhibits no plasmid (Table 3A). The chromosome of *Ahe* has an additional genomic island (GI-Ser) of about 21 kb (NT 411,561 − 432,541 in CP144673) integrated directly beneath the tRNA-Ser gene (FRA_01905, 411,471 − 411,560), not found in *Afr* and some further additional Tn-like elements across the genome (Figure S2). The GI-Ser contains two ISFw3 elements, further transposons and a putative multidrug-associated CDS (Figure S2), exhibiting low identity to proteins of *Legionella*, *Chlamydia* and *Holospora*, not present in other members of *Francisellaceae*. Genome analysis revealed that *Ahe* exhibits a cluster of genes for *myo*-inositol utilization and we could show that *Ahe* is positive for utilization of *myo*-inositol [29], for glucose-usage through glycolysis, but not through the Entner-Doudoroff or the pentose phosphate pathway [17, 29, 30].

**Table 3:**
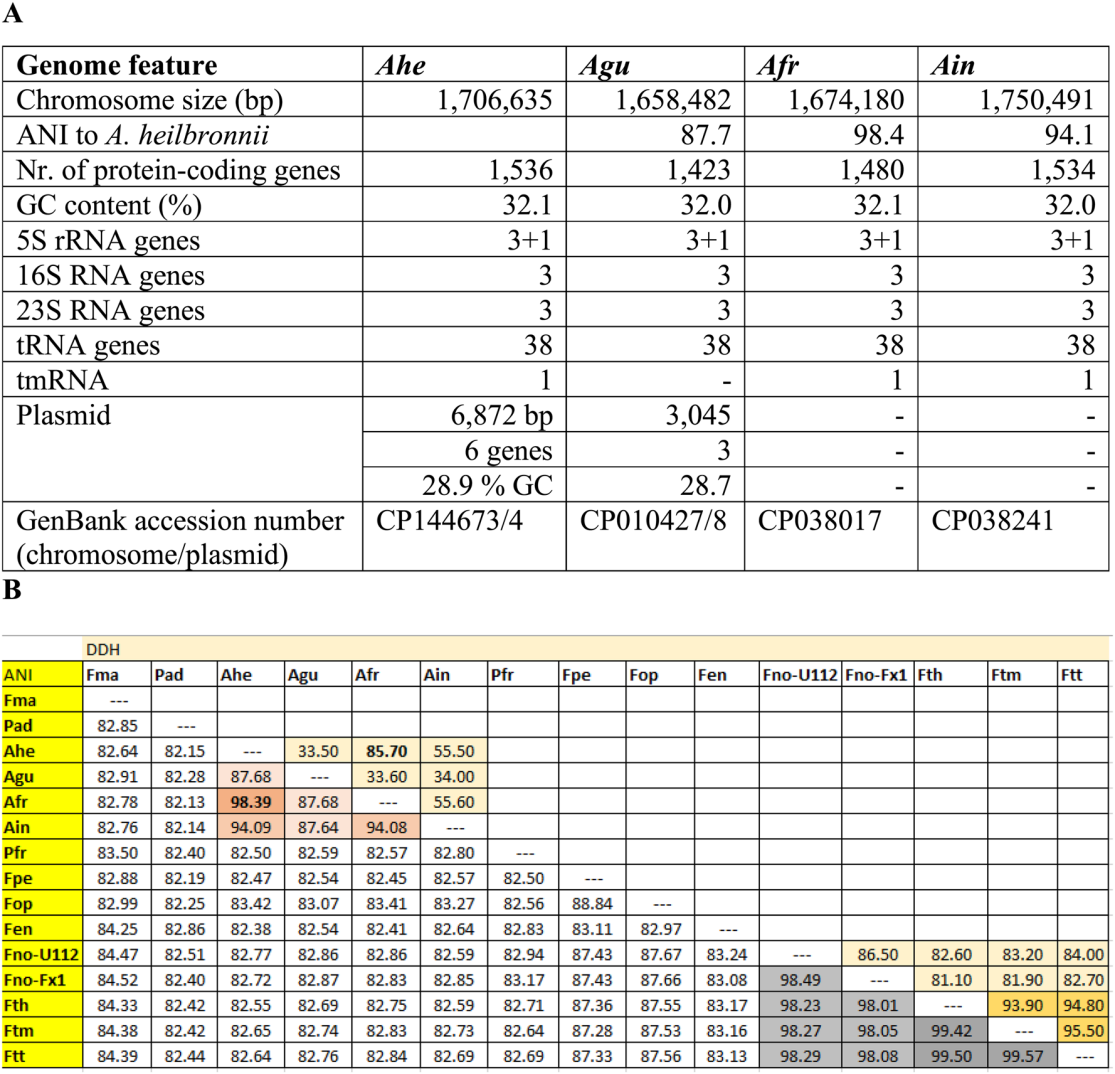
Genome features of various *Allofrancisella* genome sequences (A) and the average nucleotide identity (ANI) pairwise calculation and digital DNA-hybridization (DDH) (B)

*Ahe* *A. heilbronnii* strain W12-1067, *Agu* *A. guangzhouensis* strain 08HL01032^T^, *Afr* *A. frigidaguae* strain SYSU 10HL1970, *Ain* *A. inopinata* strain SYSU YG23, *Fen* *F. endociliophora*, *Fma* *F. marina*, *Fno* *F. novicida*, *Fop* *F. opportunistica*, *Fth* *F. tularensis* ssp. *holarctica*, *Ftm* *F. tularensis* ssp. *mediasiatica*, *Ftt* *F. tularensis* ssp. *tularensis* SchuS4, *Pad* *Parafrancisella adeliensis*, *Pfr* *Pseudofrancisella frigiditurris*

Genome comparison with other genomes of *Francisellaceae* revealed that the genus *Allofrancisella* seems to be the only genus of *Francisellaceae* without a homolog of the FPI island. Instead, it exhibits an alternative FPI [16], which is also present in *F. novicida* encoding a second putative alternative T6SS (aT6SS) in addition to the FPI encoded T6SS [31, 32]. The whole genome ANI analysis showed that the German strain *Ahe* showed ANI values of 98%, 94% and 87% to the Chinese strains *Afr*, *A. inopinata* and *A. guangzhouensis*, respectively (Table. 3B), indicating that *Ahe* belongs to genus *Allofrancisella*. An ANI value of 98.4% to *Afr* may indicate *Ahe* as a subspecies of *Afr*. However, because of the observed differences in phenotypic characteristics and chemotaxonomy (see below), *Ahe* could be classified as a new species of *Allofrancisella*. Phylogenetic analysis (Neighbor Joining method) of different whole genomes of *Francisellaceae* corroborate *Ahe* to be part of the genus *Allofrancisella*, closest related to strain *Afr* (Fig. 1).

Furthermore, the LPS of *Ahe* is significantly different to that of *Afr*, indicated by the low identity of gene products involved in LPS biosynthesis of *Ahe* on the nucleotide level in comparison to that of *Afr* (Table S1) and by the fact that an anti-*Ahe*-LPS antibody did not show any cross-reactivity with the LPS of *Afr*, *A. inopinata* or *A. guangzhouensis* (Figure S3).

### Phenotypic characteristics

*Ahe* W12-1067 is positive for valine arylamidase, trypsin and acid phosphatase, but negative for alkaline phosphatase, lipase, and α/β glucosidase (Table 4). In contrast, *Afr* is negative for valine arylamidase and trypsin, but positive for alkaline phosphatase [3].

**Table 4:** Results of API Zym for *Ahe* strain W12-1067

API ZYM (6 h, 37 °C)	Ahe W12-1067
Alcaline phosphatase	0
Esterase	3
Esterase lipase	3
Lipase	0
Leucine-arylamidase	3
Valine-arylamidase	1
Cystine-arylamidase	0.5
Trypsin	1
Chymotrypsin	0
Acid phosphatase	1
Naphtol-AS-BI-phosphohydrolase	0.5
α-galactosidase	0
β-galactosidase	0
β-glucuronidase	0
α-glucosidase	0
β-glucosidase	0
N-acetyl-β-glucosaminidase	0
α-mannosidase	0
α-fucosidase	0

reading scale according to the manufacturer (nanomole of substrate hydrolyzed)

0 = 0 nanomole, 1 = 5 nanomoles, 2 = 10 nanomoles, 3 = 20 nanomoles

In recent publications we can demonstrate that strain W12-1067 is able to utilize glucose, glycerol, *myo*-inositol, alanine and serine in a growth-phase dependent manner. Glucose is mainly metabolized through glycolysis, but not through the Entner-Douderoff pathway. Carbon flux from glycerol and serine is less active and different from *F. tularensis* [29, 30, 33]. In addition, *Ahe* showed a significantly different growth in medium T at 37 °C in comparison to *Afr* and other *Allofrancisella* strains (Figure S4).

The respiratory quinone of *Ahe* is ubiquinone-8 and the lipid profile consists of phosphatidylethanolamine, phosphatidylcholine, phosphatidylglycerol, and diphosphatidylglycerol (Figure. S5) including two prominent additional spots of glycolipids when compared to *Afr* ([3] and Figure S5). The major fatty acids of *Ahe* are C_10:0_, C_18:0_ 3-OH, C_18:1_ *w9c* and C_14:0_ (Table 5, Figure S6). The cellular fatty acid composition was significantly different between *Ahe* and *Afr* (C_10:0_, C_10:0_ 2OH, and C_18:0_ 3OH, see Table 5), confirming that *Ahe* can be considered as a new species.

**Table 5:** Relative cellular fatty acid composition (%) of *Ahe* and *Afr* strains (SYSU 10HL1970^T^, 10HP82-10, 10HL1938, 10HP457) *A. inopinata* SYSU YG23^T^ (*Ain*) and *A. guangzhouensis* 08HL01032^T^ (*Agu*)

Fatty acid (>1%)	Ahe W12-1067	Afr strains*	Ain*	Agu*
10:0	15.8	19.5–21.5	14.3	20.5
10:0 2OH	0.5	5.1–6.7	7.9	3.0
12:0	1.1	1.4–1.5	1.6	1.3
14:0	11.3	6.7–7.7	6.6	9.4
16:0	8.7	6.2–9.2	17.8	18.2
16:0 3OH	9.1	8.5–9.7	8.6	8.2
18:1 w9c	12.2	9.1–10.9	9.9	10.6
18:0	8.9	7.7–10.3	2.0	3.6
18:0 3OH	12.6	18.2–20.5	22.2	17.5
20:1 w11c	2.0	n.d	n.d	n.d
20:0	3.2	2.7–3.5	3.0	2.8

* data from [3]

Growth of strain W12-1067 was not inhibited in the presence of ampicillin, penicillin G, piperacin/tazobactam, vancomycin and fosfomycin, zones of inhibition were observed in the presence of ciprofloxacin, levofloxacin, gentamicin, tetracyclin and trimethoprim-sulfamethoxazole (Figure S7). More detailed phenotypic characteristics are given in the species description.

In consideration of the above-mentioned differences and with the distinct geographical origins of *Ahe* and *Afr* (Germany and China), we classified isolate W12-1067 as a new species of *Allofrancisella*, named as *A. heilbronnii* after its initially finding location [16].

### General features of plasmid pFW12 identified in *Allofrancisella heilbronnii*

While resequencing and reanalyzing of the genome sequence of *Ahe*, an extrachromosomal sequence was found. The complete nucleotide sequence of this contig has a size of 6,872 bp and a GC-content of 28.9%, which is lower than for the chromosome of *Ahe* (32.1%), and it partly corresponds to contig_71 as previously published [16]. To verify the presence of a plasmid and its circularization, extrachromosomal DNA was extracted from *Ahe* and used in PCR analysis with primers pointing outwards of contig_71 (Contig_71 F/R, Table 1, data not shown). PCR analysis confirmed that this sequence is circular and the extrachromosomal plasmid was designated as pFW12.

**Fig. 2:**
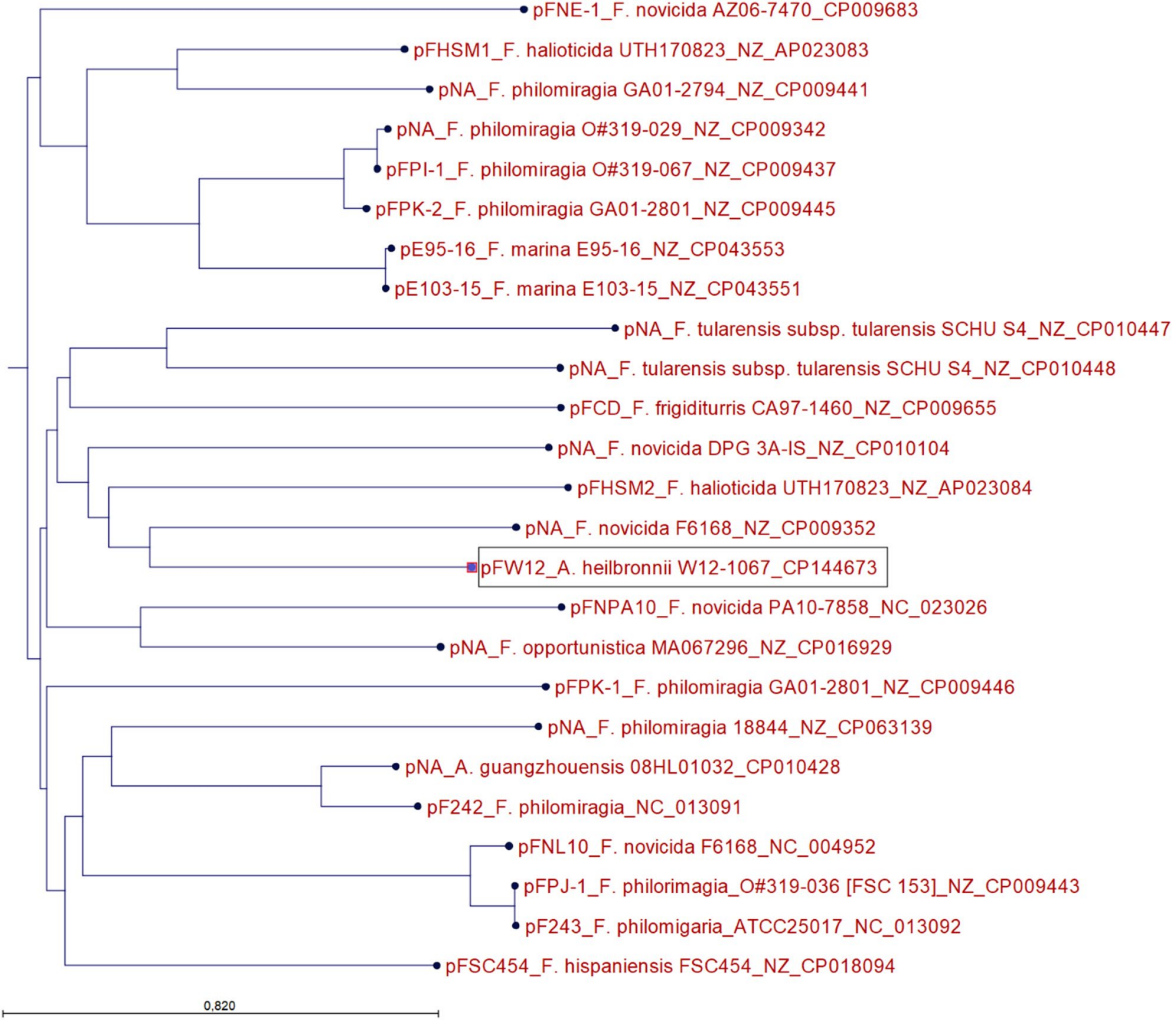
Phylogenetic tree of *Francisellaceae* plasmids. Plasmid DNA sequences were aligned in CLC Genomics Workbench using ClustalW (total length: 101341 bp) and a Neighbor Joining tree was generated (Jukes-Cantor with Bootstrap = 100 Replicates). The tree shows that the pFW12 plasmid obtained from strain W12-1067 is not closely related to *A. guangzhouensis* plasmid; rather, it is more closely related to plasmids obtained from *F. novicida* for example. The plasmid name (pNA indicates unnamed plasmids), the *Francisellaceae* strain and the accession number are given

**Fig. 3:**
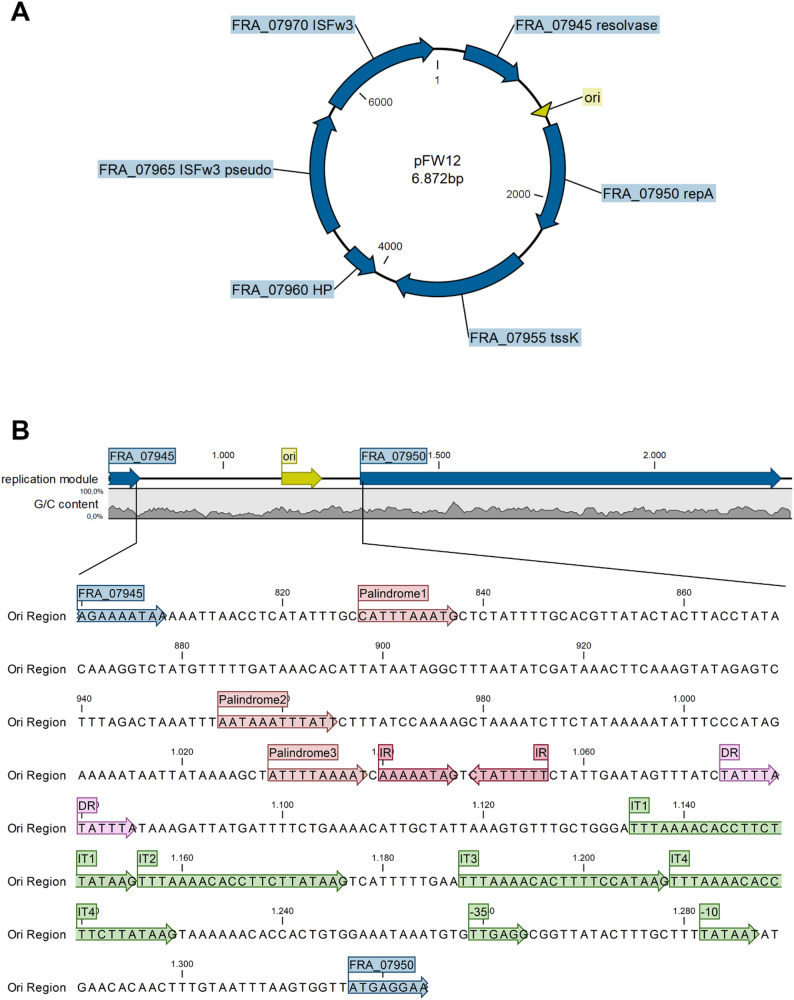
Plasmid map and the replication module of pFW12. **A**: Overview of pFW12. The plasmid comprises of 6,872 bp and six open reding frames (FRA_07945 (resolvase), FRA_07950 (*repA*), FRA_07955 (*tssK*, *iglD*), FRA_07960 (hypothetical protein [HP]), FRA_07965 (ISFw3 pseudo), FRA_07970 (ISFw3), which are indicated by blue arrows. The putative origin of replication (ori) is indicated by a yellow triangle. **B**: Replication module of pFW12. The sequence upstream of the repA gene contains four direct repeats (iterons, IT1-IT4, green), AT-rich inverted repeats (IR, dark red and palindrome 1–3, red) as well as short AT-rich direct repeats (DR, pink). The putative − 10 and − 35 boxes are highlighted in light green. The primer binding sites, which were used to generate pFW12∆ori, are indicated in purple

Phylogenetic analysis of the plasmid revealed that pFW12 is more closely related to plasmids found in *F. novicida* (DPG 3A-IS) and *F. hispaniensis* (pFSC454) than to other plasmids found in *Allofrancisella* for instance (08HL01032, see Fig. 2). The plasmids of *F. novicida* and *F. hispaniensis* are rather larger plasmids (pFSC454: 16 kb with 13 genes; Fno DPG_3A-IS: 41.9 kb with 42 genes) compared to pWF12 which has a size of 6,872 bp. pFW12 possesses six open reading frames (ORF) which constitute 74% of the whole plasmid sequence. The genetic map of pFW12 is shown in Fig. 3A and the ORFs are listed in Table 6. In silico sequence analysis revealed the presence of a *repA (FRA_07950)*, *iglD (FRA_07955*,* tssK)*, resolvase/serine recombinase (FRA_07945), two ISFw3 genes (FRA_07965-70) and one gene coding for a hypothetical protein (FRA_07960). Most of the plasmid-encoded proteins show a high percent identity to proteins found in *A. guangzhouensis* like RepA and IglD, but the serine recombinase shows a great percent identity to *F. novicida* (90.76%) and the hypothetical protein to a chromosomal gene of *Afr* (89.87%, see Table 6).

**Table 6:** Open reading frames of pFW12

ORF FRA_	Position	Length (bp)	Strand	aa	BLASTP	Putative function	Species	PerID (%)
07945	254.808	555	+	185	Recombinase family protein	Recombination	*Fno*	91
07950	1317.2294	978	+	326	Replication initiation protein	Replication	*Agu*	97
07955	2630.3826	1197	+	399	TssK	T6SS	*Agu*	89
07960	4030.4332	303	-	101	Hypothetical protein	-	*Afr*	90
07965	4574.5554	981	+	327	IS481-like element ISFw3 family transposase, pseudogene	Integrase, transposase	*Agu*	100
07970	5758.6837	1080	+	360	IS481-like element ISFw3 family transposase	Integrase, transposase	*Agu*	100

*ORF* Open reading frame, *Fno* *Francisella novicida*, *Agu* *Allofrancisella guangzhouensis*, *Afr A. frigidaquae*

**Fig. 4:**
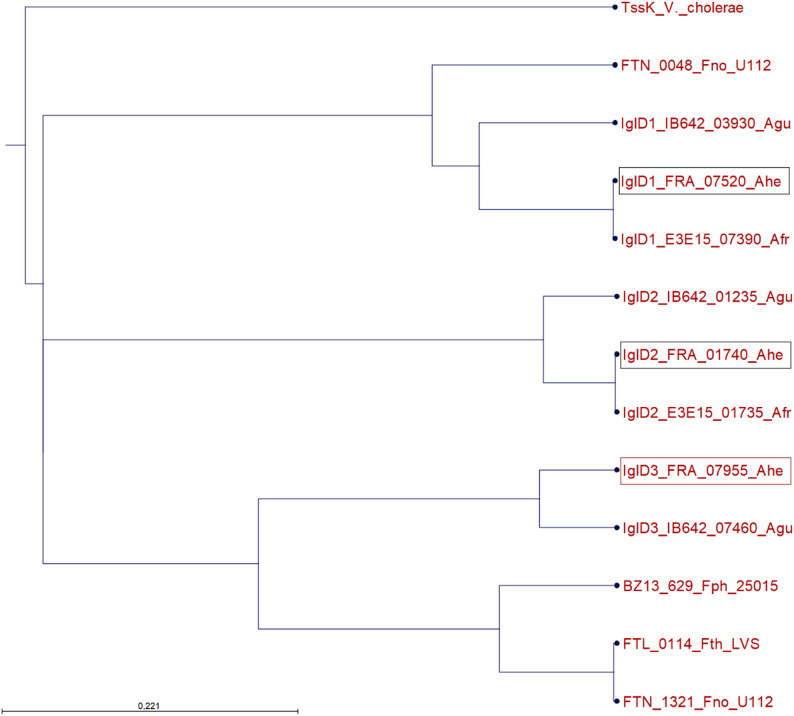
Phylogenetic relation of different IglD (TssK) proteins in *Francisellaceae*. The phylogenetic tree shows that the pFW12-encoded IglD protein (IglD3, FRA_07955) from *Ahe* is closely related to the chromosomal FPI-encoded IglD protein found in all *Francisella* species, such as *F. tularensis* ssp. *holarctica* (FTL_0114). The tree was generated based on a ClustalO alignment with the canonical TssK from *V. cholerae* (PWG95785, QFKZ01000006), the FPI-encoded IglD proteins from *F. tularensis* ssp. *holarctica* (Fth) LVS (FTL_0114, NC_007880), *F. novicida* (Fno) U112 (FTN_1321, NC_008601) and *F. philomiragia* (Fph) 25,015 (BZ13_629, CP010019); as well as the Fno-FPI IglD FTN_0048 from *F. novicida* (Fno) U112 (NC_008601) and the “IglD” protein found in *Allofrancisella*: FRA_07520, FRA_01740 and plasmid-encoded FRA_07955 from *A. heilbronnii* (Ahe, CP144673); IB642_03930, IB642_01235, IB642_07460 from *A. guangzhouensis* strain 09HG994 (JACVKI000000000); E3E15_07390 and E3E15_01735 from *A. frigidaquae* (CP038017)

In *Ahe* three IglD proteins are found: one is located on pFW12 (FRA_07955) and the other two are found in the FPI-like genomic islands (FRA_01740, FRA_07520 [16]),. The *Francisella iglD* gene encodes a baseplate protein of the T6SS basal complex that shares structural homologies with the canonical TssK of *E. coli* [34]. IglD has been shown to be essential for *Francisella* replication in macrophages and for the full virulence in mice [19, 31]. Interestingly, the plasmid-encoded IglD protein from *Ahe* is more related to the chromosomal FPI-encoded IglD protein found in all *Francisella* species, like *F. tularensis* ssp. *holarctica*, *F. novicida* and *F. philomiragia*, when compared to the chromosomal encoded IglD-like proteins of the putative aT6SS in *Ahe*, as shown by phylogenetic relation analysis (Fig. 4). In contrast, in the closely related *Afr* only two IglD proteins exits, which show less similarities to the FPI-IglD found in *F. tularensis* ssp. *holarctica*, *F. novicida* and *F. philomiragia*. In *A. guangzhouensis* strain FSC1085 an IglD is found which show a great similarity to the pFW12-encoded IglD in *Ahe*. The significance of a plasmid-encoded IglD protein remains unclear and should be addressed in future studies, as should the alternative T6SS in *Ahe*.

Interestingly, the two plasmid-encoded ISFw3 genes are not identical (Figure S1), as seen for the chromosome (see above). The FRA_07970 gene is 1080 bp in length coding for a protein with a size of 360 aa. In contrast, the pseudo-ISFw3 gene (FRA_07965) is 981 bp in length encoding a putative 327 aa-sized protein.

FRA_07945 encodes for a putative resolvase (Table 6), exhibiting motifs for a serine recombinase, an invertase (SpoIVCA superfamily) and excisionase (rec_xisF superfamily) and shows homolog proteins in *Afr*, *F. halioticida* and *Legionella* spp. Thus, this protein seems to be a DNA recombinase, probably involved in DNA inversion, integration or excision. Furthermore, the plasmid-encoded resolvase was identified in a virulence screening test using a Tn5 mutant bank of *Ahe* W12-1067 and *Acanthamoeba lenticulate* as a host (so-called Scatter Screen, see reference [17]). The Tn5 transposase was inserted into the resolvase gene in clone Sc#64. Compared to the wild-type *Ahe* W12-1067, the survival of Sc#64 was found to be slightly reduced in *A. lenticulata* and *Drosophila* S2 cells (data not shown). Due to the antibiotic resistance marker of the Tn5 insertion construct, the plasmid of Sc#64 (pFW12-Sc64) was used for further experiments (see below).

FRA_07960 encodes a hypothetical protein with 90% percent identity to a hypothetical protein found in *Afr*. However, no other significant homologies could be identified. Using the HHpred server, which considers protein structure and function to identify protein relationships, a Domain of Unknown Function (DUF4468) with a TATA-binding protein-like fold was identified, but with a low probability of only 44%. In conclusion, the majority of pFW12-encoded gene products appear to be involved in DNA replication and DNA modelling (integration, excision etc.). To date, no pFW12-mediated function has been observed, leading to the assumption that pFW12 may be a cryptic plasmid.

### Organisation of the replication system of pFW12

In Gram-negative bacteria, the replication system of theta mode replicating plasmids comprises a gene encoding a replication initiation protein (Rep) and an upstream origin of replication (*ori*). The plasmid pFW12 was found to contain both elements (Fig. 3). The putative *repA* gene of pW12 encodes a protein of 326 amino acids (aa; FRA_07950 with 978 nucleotides). It exhibits highest degree of aa sequence identity with the replication initiation protein from *A. guanghzouensis* (97%, see Table 6). Further, the *Ahe* RepA protein exhibits general characteristics of rep proteins including a leucine zipper motif for protein-protein interactions and helix-turn-helix motifs for DNA-binding.

The *ori* sequence usually possesses several distinct characteristics, including a relatively low GC-content and the presence of specific direct repeats, named iterons, which are recognised by the plasmid-encoded rep protein. These iterons are arranged as tandem repeats (4–7 repeats) with a length of 20–22 bp. In pFW12, four 21-bp iterons (5’-TTTAAAACACCTTCTTATAAG-3’, IT1-IT4) were identified, which are located 87 bp upstream of the predicted start codon of *repA* (ATG, see Fig. 3B). IT1, IT2 and IT4 are identical, while IT3 exhibits four mismatches compared to the others (5’-TTTAAAACAC*T*TT*TCC*ATAAG-3’). Slightly upstream, AT-rich direct repeats (DR 5’-TATTTA-3’) and AT-rich inverted repeats were found (IR 5’-AAAAATAG-3’), of which three form a palindromic sequence (palindrome1: 5’-CATTTAAATG-3’, palindrome2: 5’-AATAAATTTATT-3’, palindrome3: 5’-ATTTTAAAAT-3’, Fig. 3).

To confirm that the iteron-containing region represents the *ori*, a pFW12 was generated lacking all four iterons. Therefore, primers were designed to amplify the entire plasmid, with the expectation of the putative origin of replication (see Fig. 3B). The native plasmid of Sc#64 (pFW12-Sc64) and the plasmid lacking both IS-elements (see above) were used as templates. The resulting PCR products were religated leading to pFW12-Sc64∆ori and pFW12-Sc64∆IS∆ori plasmids, which were transformed into *F. tularensis* ssp. *holarctica* LVS. For control purpose, the plasmid pFW12-Sc64∆IS was in parallel used for transformation (see above) and its transformation effectivity was set as 100%. In total, 1012 *F. tularensis* ssp. *holarctica* LVS pFW12-Sc64∆IS clones were obtained. In contrast, the transformation of both plasmids lacking the putative *ori*, pFW12-Sc64∆ori and pFW12-Sc64∆IS∆ori, resulted in only a few clones: 267 *F. tularensis* ssp. *holarctica* LVS pFW12-Sc64∆ori clones and 115 *F. tularensis* ssp. *holarctica* LVS pFW12-Sc64∆IS∆ori clones. The transformation effectivity was calculated as 26% and 11%, respectively. Then, five clones each were tested by PCR and Sanger sequencing for the presence of the *ori* sequence. All tested clones revealed the presence of the native pFW12 *ori* region leading to the assumption that the above described iteron-containing region is essentially required for the replication of the pFW12.

### Plasmid transformation and stability testing

Next, we aimed to find out, if the plasmid pFW12 is transferable and stable in other strains or species of the family *Francisellaceae*, to investigate if the plasmid can be used as a new vector for *Francisellaceae* strains. As mentioned above, using the Scatter Screening method, a clone (Sc#64) was identified where the Tn5 transposase was inserted into the resolvase (see above and [17]). The Sc#64 showed a slightly reduced survival in *A. lenticulate* and *Drosophila* S2 cells (data not shown). Since the plasmid of Sc#64 was marked with a kanamycin resistance gene, this plasmid pFW12-Sc64 was further used for the stability testing experiments.

In addition, we wanted to check if pFW12-Sc64 is also transferable and stable when both ISFw3 elements are absent. Therefore, a plasmid was generated lacking both ISFw3 elements by using the primer Contig 71 F3 and Contig 71 R3, which lack would be better to use it as a general vector. The resulting PCR product was religated and used for DNA transformation using electroporation into *Ahe* wild-type, *F. tularensis* ssp. *holarctica* LVS, *F. novicida* U112, *A. inopinata* and *F. philomiragia* 25,017. We received positive clones for all *Francisellaceae* tested, except for *F. philomiragia* 25,017. All clones were positively tested by using several PCR assays.

As a next step, we assessed if pFW12 is stable, even without antibiotic selection stress. Therefore, two clones each from LVS pFW12-Sc64 and LVS pFW12-Sc64∆IS were cultured for 12 to 24 h and subsequently passaged ten times in medium supplemented with kanamycin. Afterwards, bacteria were plated onto agar plates with and without kanamycin and tested in PCR assays for the presence of pFW12. Even after 10 passages the pFW12 plasmid was stable in *F. tularensis* ssp. *holarctica* LVS as seen in Figure S8. In conclusion, we showed that pFW12 is transferable and stable in other *Francisellaceae*; even without IFw3 elements. Therefore, no integration into the genome/ISFw3 activity is assumed so far and further experiments will demonstrate if the plasmid can be used as a vector for *Francisellaceae*.

## Conclusion

The genus *Allofrancisella* seems to be the only member of *Francisellaceae* which is negative for the genes of the major virulence island FPI, but it encodes a putative alternative T6SS which was also found in *F. novicida*. Phylogenetic analysis revealed that *Francisella* sp. strain W12-1067 belongs to the genus *Allofrancisella* and now was designated as *A. heilbronnii*. Although the high ANI value with *Afr*, the strain W12-1067 was designated as a separate species, due to following observations: (1) strain W12-1067 shows specific and distinct phenotypical characteristics, including a different growth behavior; (2) it exhibits a significantly different LPS structure and an anti-*Ahe*-LPS antibody showed no cross-reactivity with the LPS of *Afr*; (3) W12-1067 differed significantly from *Afr* in its cellular fatty acid composition and in its biochemical characteristics (chemotaxonomy); (4) The major ISFw3-element is not present in the genome of *Afr*.

### Description of *Allofrancisella heilbronnii* sp. nov

The isolate W12-1067 was named *Allofrancisella heilbronnii* strain W12-1067, referring to the original isolation of the strain from a water reservoir of a cooling tower in the city of Heilbronn, Germany. The description of the strain is originally published by Rydzewski et al. (2014) as *Francisella* sp. strain W12-1067 [16]. Strain *Ahe* W12-1067 was deposited in the German Collection of Microorganisms and cell cultures (DSMZ, DSM 118443).

It grows well on MTKH, GVPC, CHA, Neisseria and Choc-Vitox plates and media AYE and medium T. L-cysteine within BCYE agar plates stimulates the growth of *Ahe*. Its growth optimum is approximately 30 °C and it can grow in the presence of 4–5% NaCl. The strain is positive for esterase, esterase lipase, leucine arylamidase, valine arylamidase, trypsin, acid phosphatase and negative for alcaline phosphatase, lipase, chymotrypsin, α- and β-galactosidase, β-glururonidase, α- and β-glucosidase, N-acetyl-β-glucosaminidase, α-mannosidase and α-fucosidase (see Table 4). The respiratory quinone is ubiquinone-8 and the lipid profile consists of phosphatidylethanolamine, phosphatidylcholine, phosphatidylglycerol, diphosphatidylglycerol and further phospho-, aminophospho-and glycolipids (Figure S5). The major fatty acids of *Ahe* are C_10:0_, C_18:0_ 3-OH, C_18:1_ *w9c* and C_14:0_ (Figure S6). There was no inhibition of growth of *Ahe* in the presence of ampicillin, oxacillin, penicillin G, ceftazidime, ceftriaxone, piperacin/tazobactam, vancomycin, colistin sulphate, clindamycin, fosfomycin, but zones of inhibition were visible in the presence of imipenem, ciprofloxacin, levofloxacin, gentamicin, tetracyclin, kanamycin, chloramphenicol, rifampicin and trimethoprim-sulfamethoxacole (for details, see Figure S7).

## Supplementary Information

Supplementary Material 1.

Supplementary Material 2.

## Data Availability

All data generated or analysed during this study are included in this published article [and its supplementyra information files]. The complete version of the genome (BioProject accession number PRJNA214898, BioSample: SAMN02315612) has been deposited to GenBank under the accession number CP144673 (chromosome) and CP144674 (plasmid).
