# Supplementary material for: Complete genome and plasmid sequence, and chemotaxonomic analysis of Francisella sp. strain W12-1067, now designated as Allofrancisella heilbronnii
Source: BMC Microbiol. 2025 Dec 9;25:793. doi: 10.1186/s12866-025-04600-5 (PMC12701580; doi:10.1186/s12866-025-04600-5)
Supplement: Supplementary file 1 — Supplementary Material 1. [file 12866_2025_4600_MOESM1_ESM.pptx]

## Slide 1
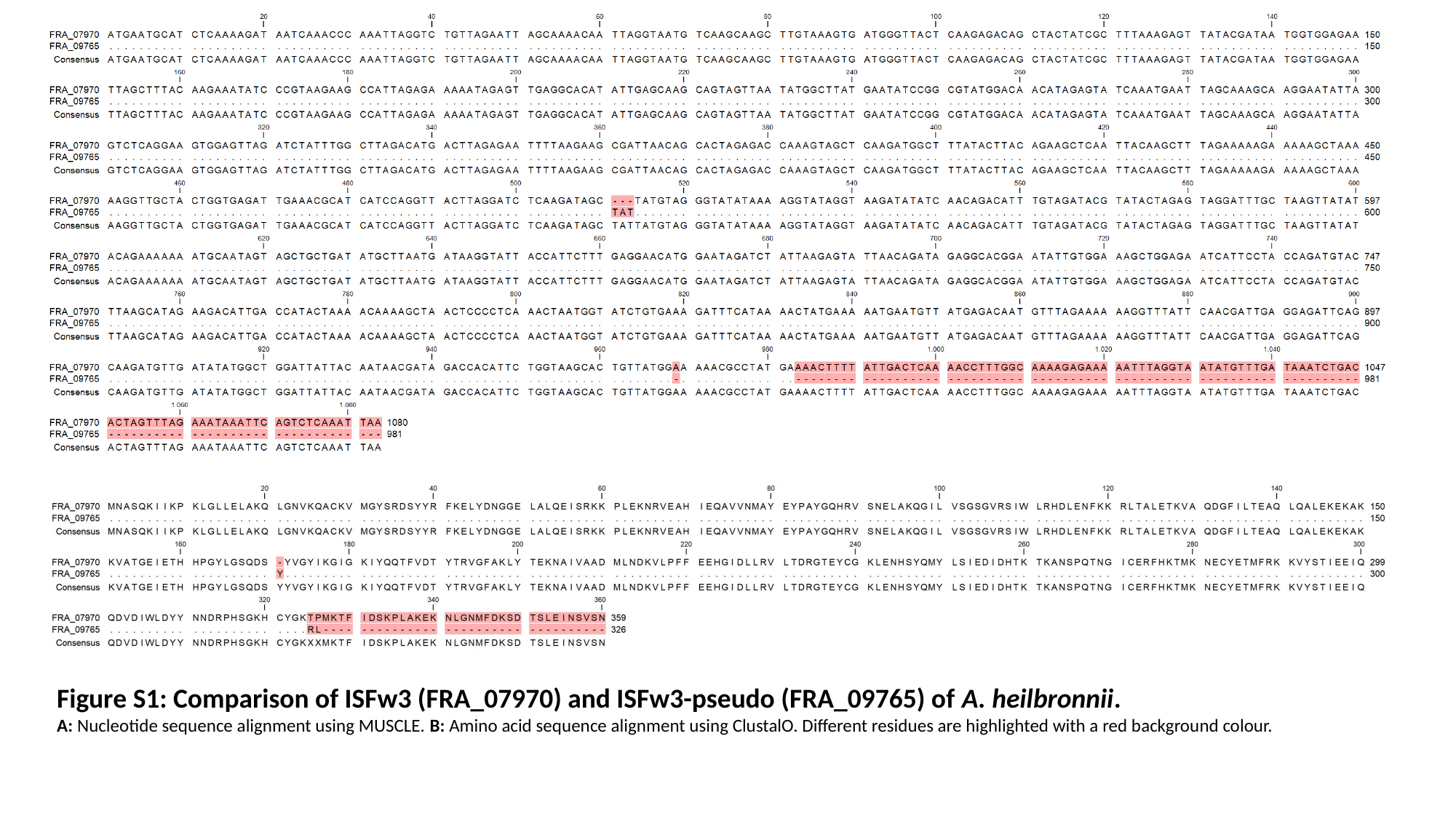

Figure S1: Comparison of ISFw3 (FRA_07970) and ISFw3-pseudo (FRA_09765) of A. heilbronnii.
A: Nucleotide sequence alignment using MUSCLE. B: Amino acid sequence alignment using ClustalO. Different residues are highlighted with a red background colour.

## Slide 2
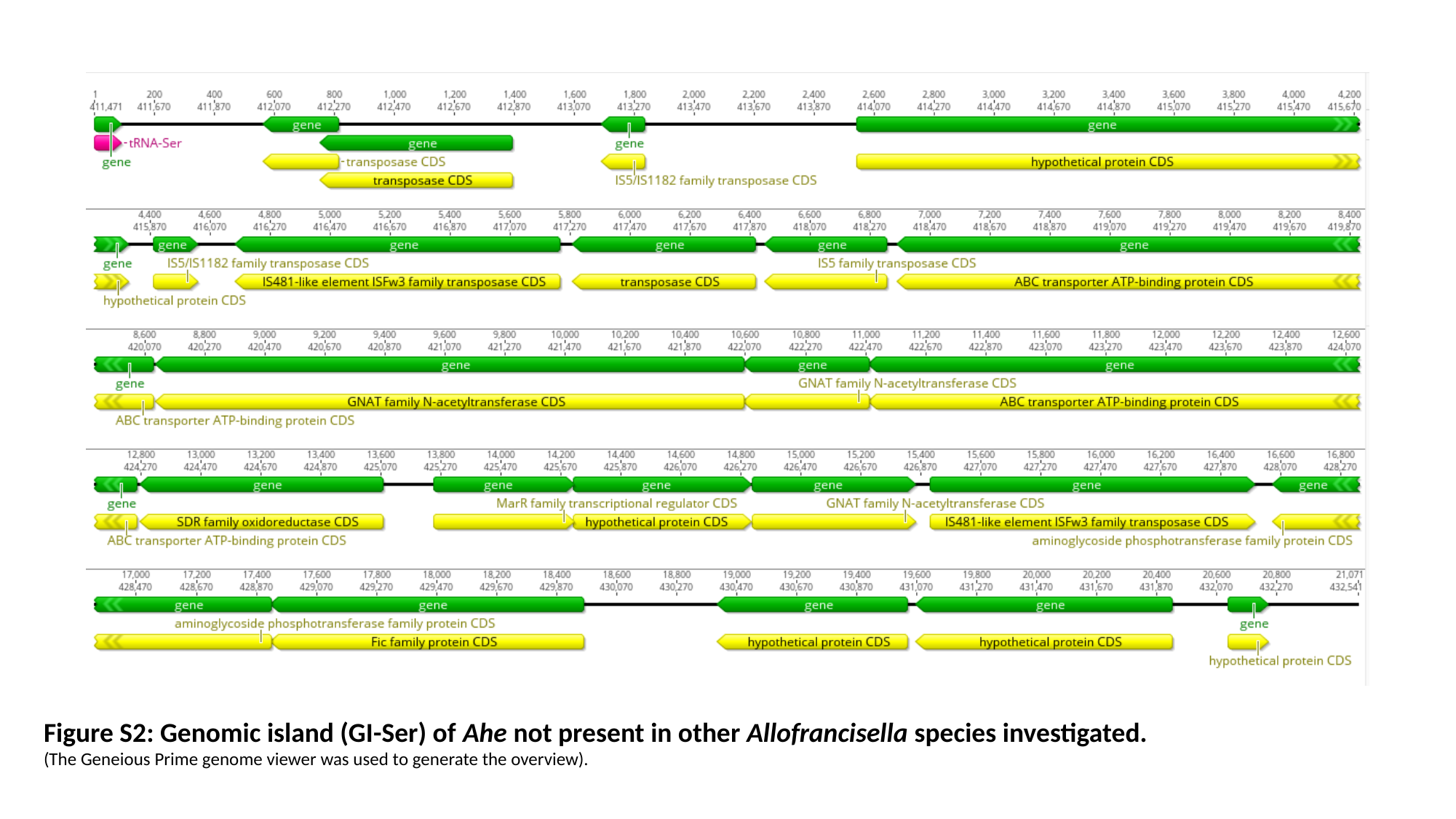

Figure S2: Genomic island (GI-Ser) of Ahe not present in other Allofrancisella species investigated.
(The Geneious Prime genome viewer was used to generate the overview).

## Slide 3
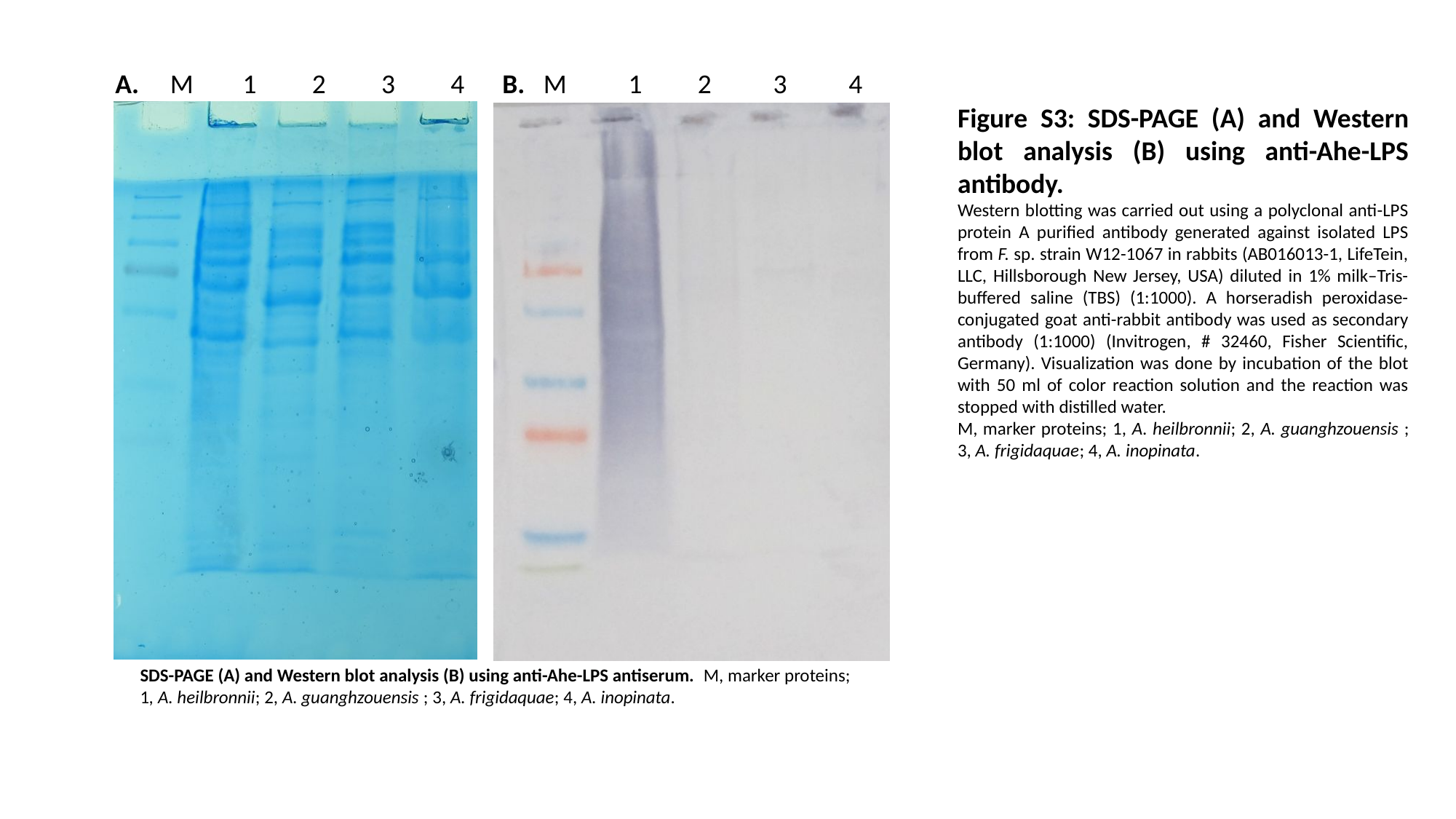

A. M 1 2 3 4 B. M 1 2 3 4
Figure S3: SDS-PAGE (A) and Western blot analysis (B) using anti-Ahe-LPS antibody.
Western blotting was carried out using a polyclonal anti-LPS protein A purified antibody generated against isolated LPS from F. sp. strain W12-1067 in rabbits (AB016013-1, LifeTein, LLC, Hillsborough New Jersey, USA) diluted in 1% milk–Tris-buffered saline (TBS) (1:1000). A horseradish peroxidase-conjugated goat anti-rabbit antibody was used as secondary antibody (1:1000) (Invitrogen, # 32460, Fisher Scientific, Germany). Visualization was done by incubation of the blot with 50 ml of color reaction solution and the reaction was stopped with distilled water.
M, marker proteins; 1, A. heilbronnii; 2, A. guanghzouensis ; 3, A. frigidaquae; 4, A. inopinata.
SDS-PAGE (A) and Western blot analysis (B) using anti-Ahe-LPS antiserum. M, marker proteins;
1, A. heilbronnii; 2, A. guanghzouensis ; 3, A. frigidaquae; 4, A. inopinata.

## Slide 4
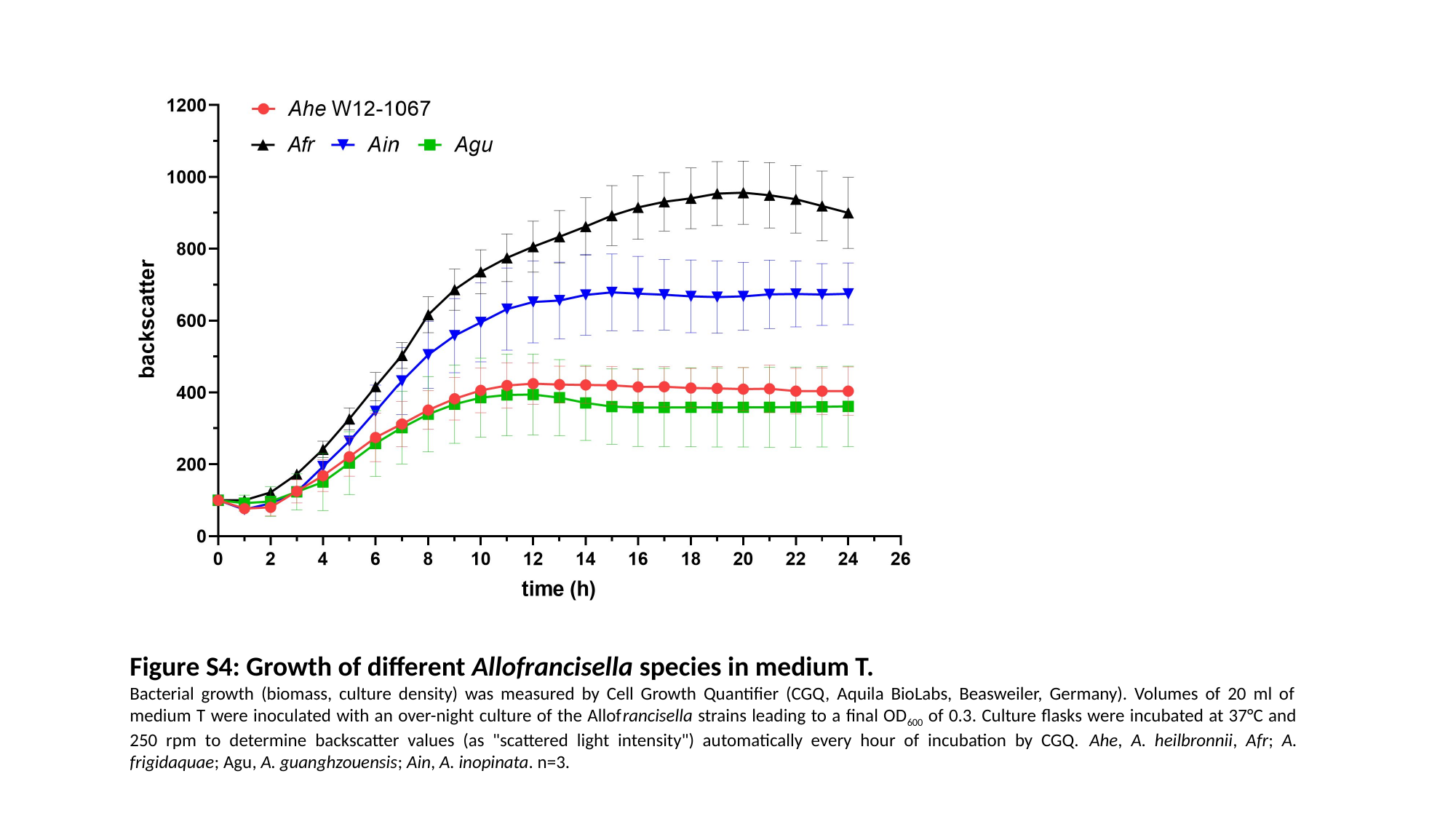

Figure S4: Growth of different Allofrancisella species in medium T.
Bacterial growth (biomass, culture density) was measured by Cell Growth Quantifier (CGQ, Aquila BioLabs, Beasweiler, Germany). Volumes of 20 ml of medium T were inoculated with an over-night culture of the Allofrancisella strains leading to a final OD600 of 0.3. Culture flasks were incubated at 37°C and 250 rpm to determine backscatter values (as "scattered light intensity") automatically every hour of incubation by CGQ. Ahe, A. heilbronnii, Afr; A. frigidaquae; Agu, A. guanghzouensis; Ain, A. inopinata. n=3.

## Slide 5
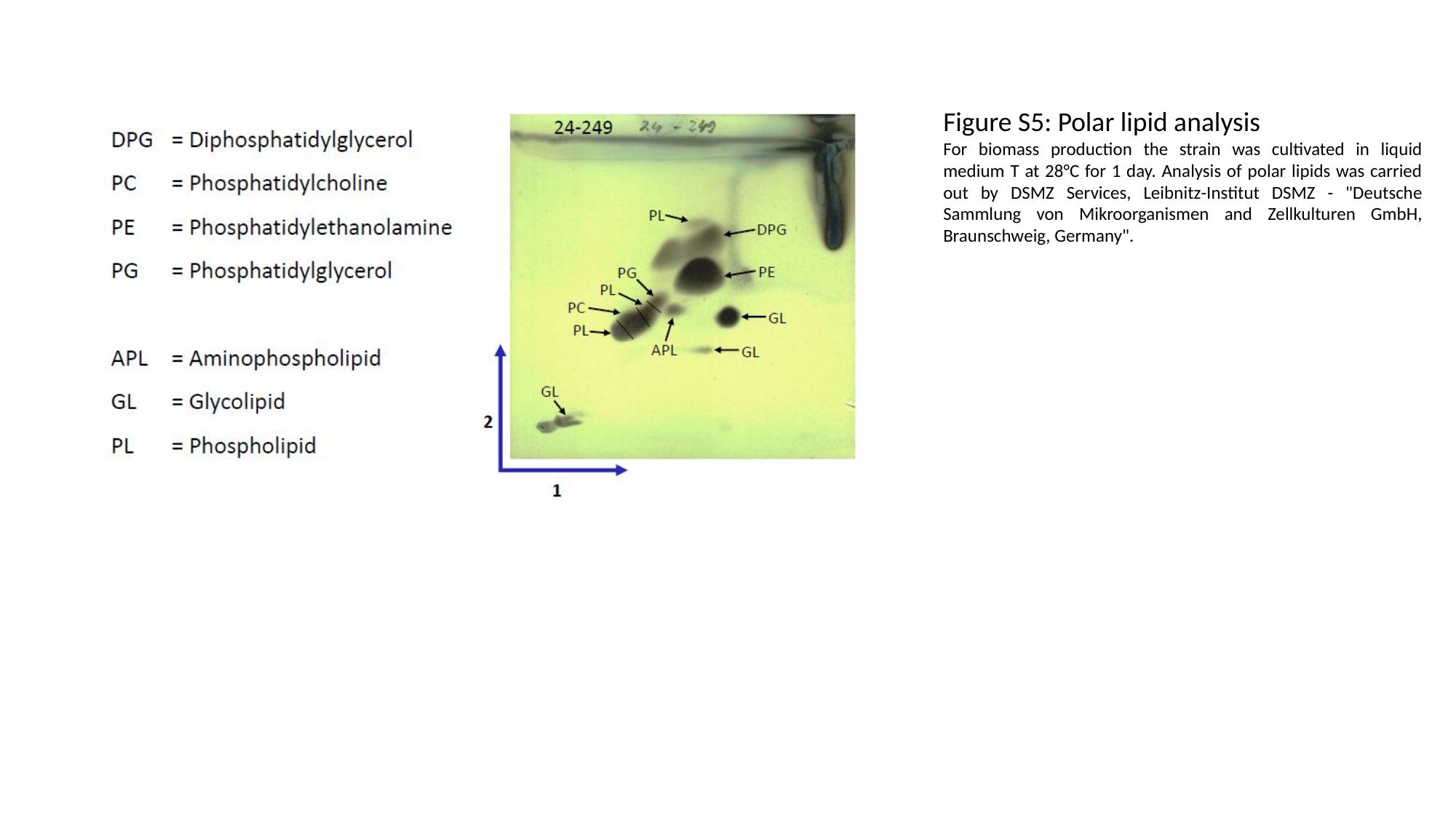

Figure S5: Polar lipid analysis
For biomass production the strain was cultivated in liquid medium T at 28°C for 1 day. Analysis of polar lipids was carried out by DSMZ Services, Leibnitz-Institut DSMZ - "Deutsche Sammlung von Mikroorganismen and Zellkulturen GmbH, Braunschweig, Germany".

## Slide 6
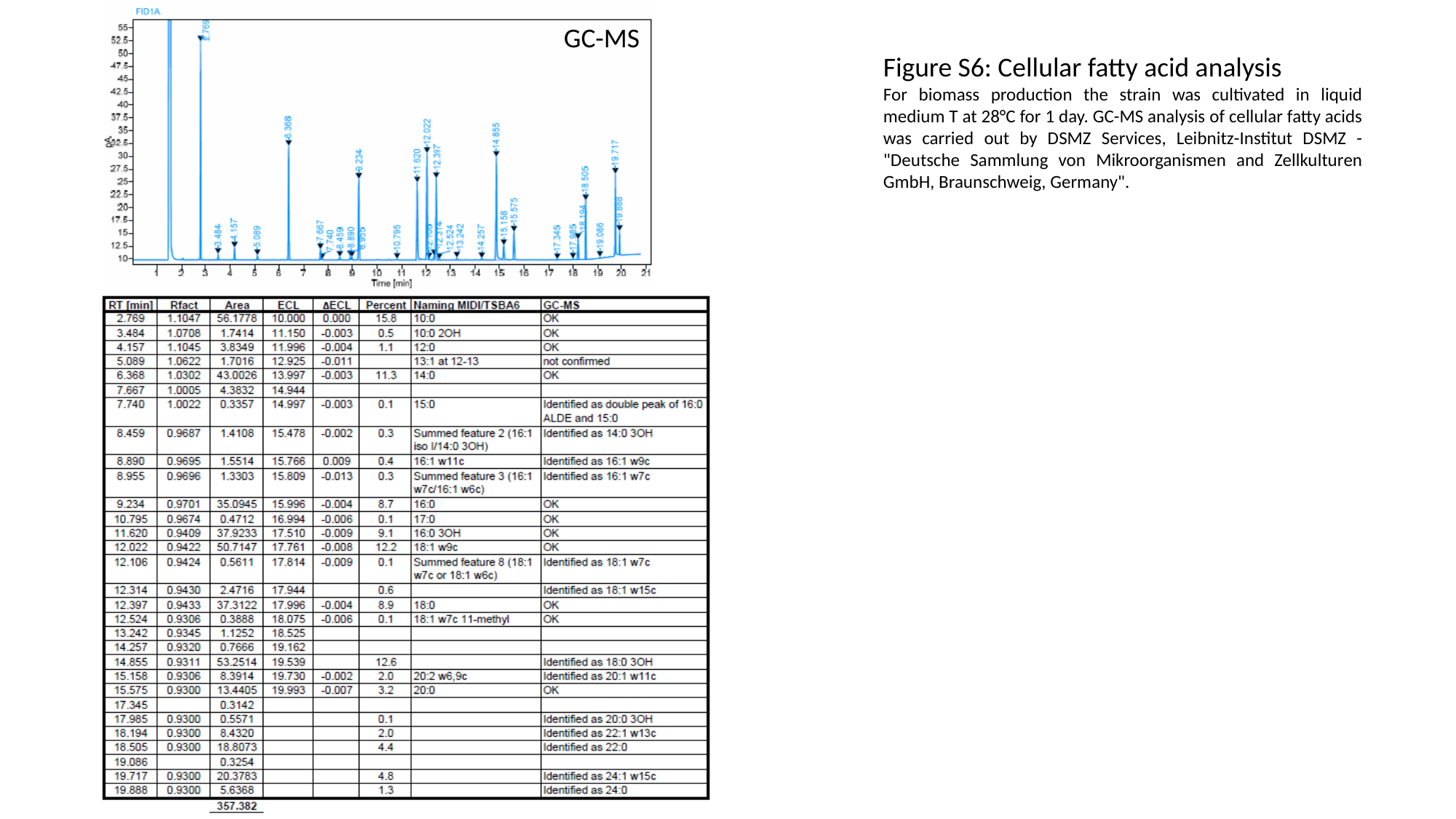

GC-MS
Figure S6: Cellular fatty acid analysis
For biomass production the strain was cultivated in liquid medium T at 28°C for 1 day. GC-MS analysis of cellular fatty acids was carried out by DSMZ Services, Leibnitz-Institut DSMZ - "Deutsche Sammlung von Mikroorganismen and Zellkulturen GmbH, Braunschweig, Germany".

## Slide 7
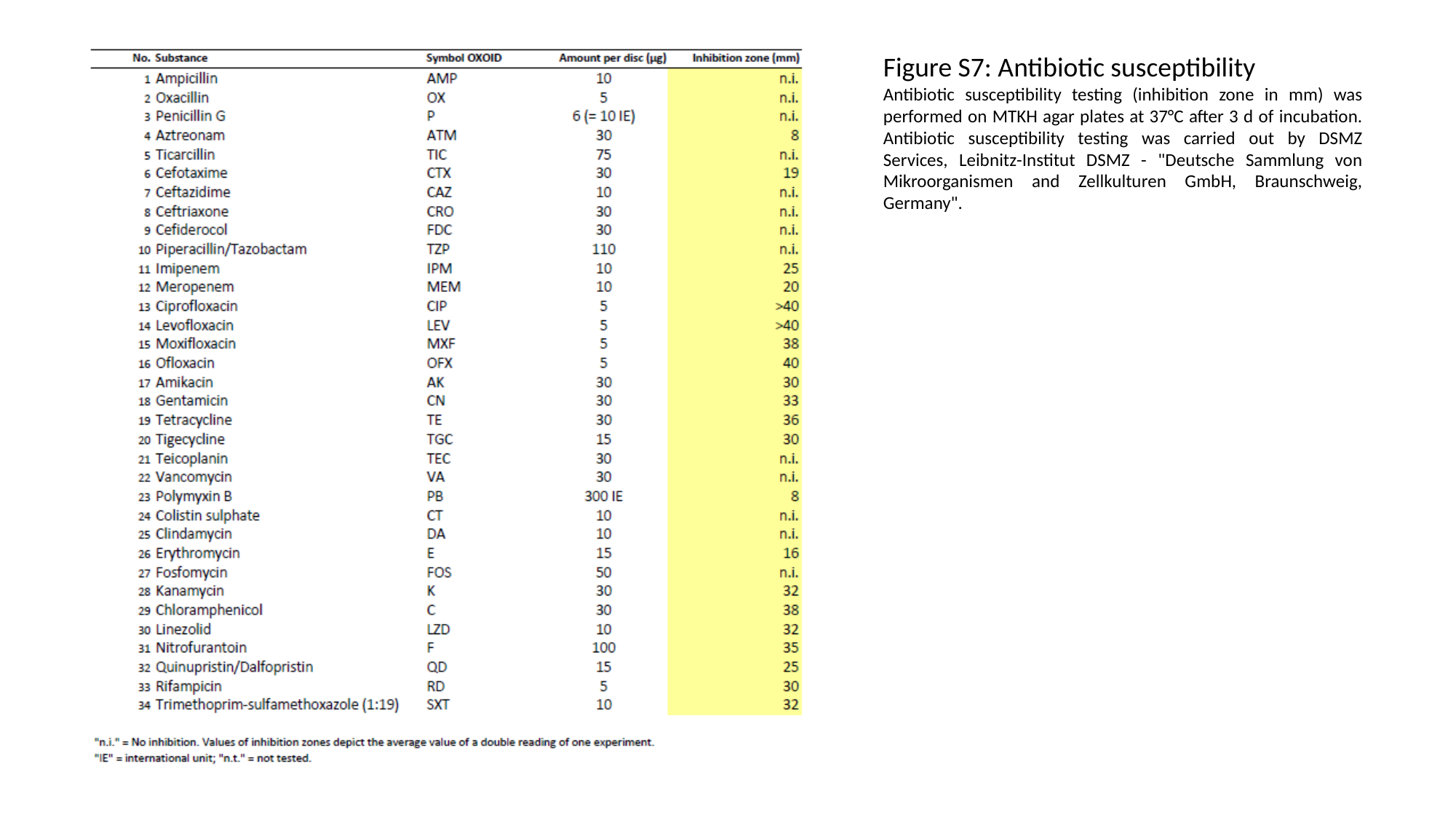

Figure S7: Antibiotic susceptibility
Antibiotic susceptibility testing (inhibition zone in mm) was performed on MTKH agar plates at 37°C after 3 d of incubation. Antibiotic susceptibility testing was carried out by DSMZ Services, Leibnitz-Institut DSMZ - "Deutsche Sammlung von Mikroorganismen and Zellkulturen GmbH, Braunschweig, Germany".

## Slide 8
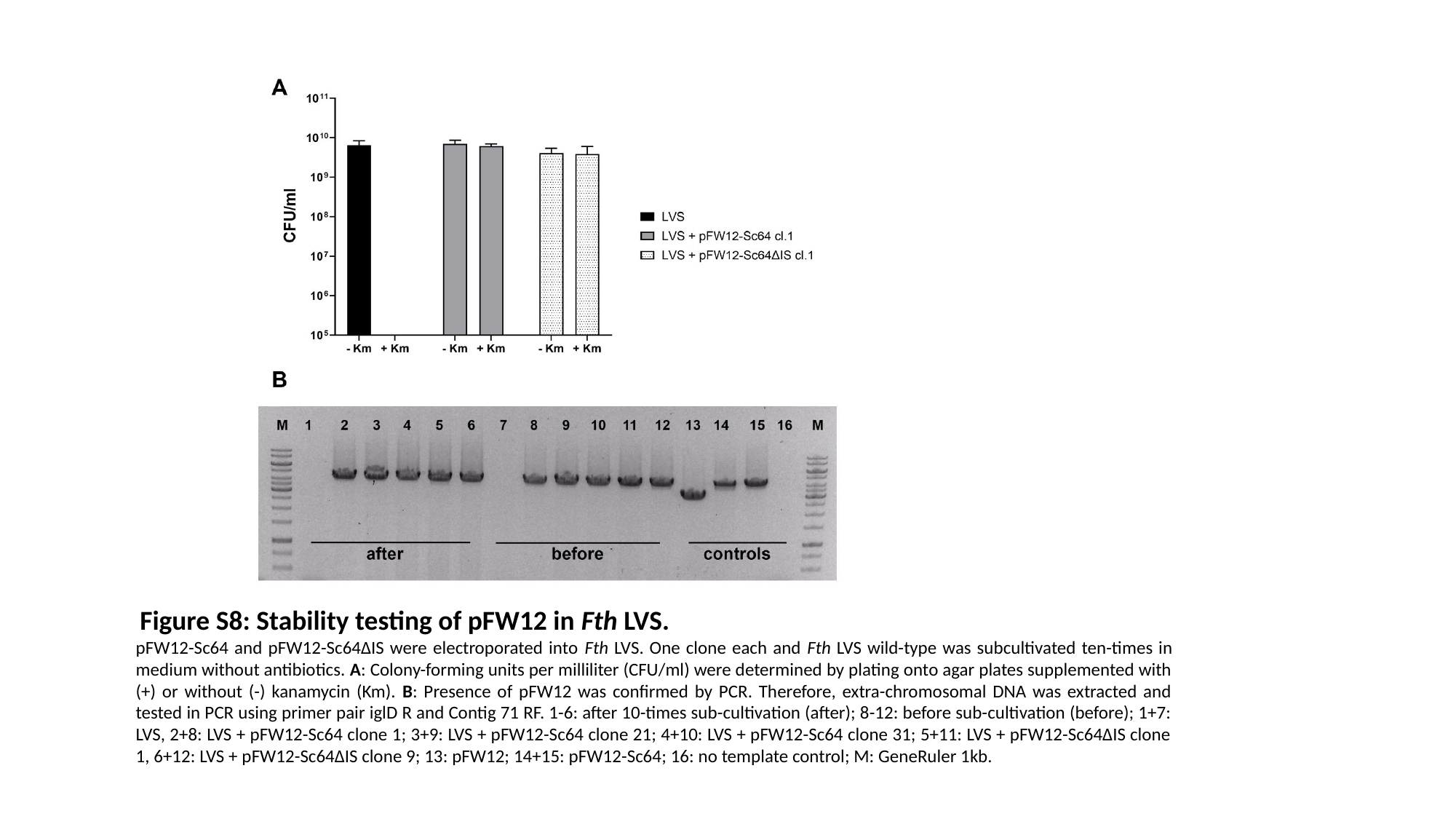

Figure S8: Stability testing of pFW12 in Fth LVS.
pFW12-Sc64 and pFW12-Sc64∆IS were electroporated into Fth LVS. One clone each and Fth LVS wild-type was subcultivated ten-times in medium without antibiotics. A: Colony-forming units per milliliter (CFU/ml) were determined by plating onto agar plates supplemented with (+) or without (-) kanamycin (Km). B: Presence of pFW12 was confirmed by PCR. Therefore, extra-chromosomal DNA was extracted and tested in PCR using primer pair iglD R and Contig 71 RF. 1-6: after 10-times sub-cultivation (after); 8-12: before sub-cultivation (before); 1+7: LVS, 2+8: LVS + pFW12-Sc64 clone 1; 3+9: LVS + pFW12-Sc64 clone 21; 4+10: LVS + pFW12-Sc64 clone 31; 5+11: LVS + pFW12-Sc64∆IS clone 1, 6+12: LVS + pFW12-Sc64∆IS clone 9; 13: pFW12; 14+15: pFW12-Sc64; 16: no template control; M: GeneRuler 1kb.
